# 2D Flower-like CdS@Co/Mo-MOF as Co-Reaction Accelerator of g-C_3_N_4_-Based Electrochemiluminescence Sensor for Chlorpromazine Hydrochloride

**DOI:** 10.3390/bios14120586

**Published:** 2024-12-02

**Authors:** Xiaowei Fan, Guping Zhang, Xiaodi Li, Yao Wang, Yi Wang, Shilei Hao, Defang Liu

**Affiliations:** Key Laboratory of Biorheological Science and Technology, Ministry of Education, College of Bioengineering, Chongqing University, No. 174, Shapingba Main Street, Chongqing 400030, China19522021380@163.com (G.Z.); 18339208382@163.com (X.L.); 18788480180@163.com (Y.W.); wangy_426986@163.com (Y.W.); shilei_hao@cqu.edu.cn (S.H.)

**Keywords:** electrochemiluminescence, CdS@Co/Mo-MOF, co-reaction accelerators, chlorpromazine hydrochloride, quenching effect

## Abstract

In this study, we have proposed an electrochemiluminescence (ECL) signal amplification system which is based on two-dimensional (2D) flower-like CdS@Co/Mo-MOF composites as a co-reaction accelerator of the g-C_3_N_4_/S_2_O_8_^2−^ system for ultrasensitive detection of chlorpromazine hydrochloride (CPH). Specifically, the 2D flower-like Co/Mo-MOF with mesoporous alleviated the aggregation of CdS NPs while simultaneously fostering reactant-active site contact and improving the reactant–product transport rate. This allowed the material to act as a novel co-reaction accelerator, speeding up the transformation of the S_2_O_8_^2−^ into SO_4_^•−^ and enhancing the cathodic ECL emission of g-C_3_N_4_. Moreover, the signal probe which was synthesized by coupling the 2D CdS@Co/Mo-MOF and graphitic carbon nitride (g-C_3_N_4_) achieved the generation of SO_4_^•−^ in situ and reduced energy loss. The results confirmed that the ECL signal was enhanced 6.2-fold and stabilized by CdS@Co/Mo-MOF. Based on the extremely strong quenching effect of chlorpromazine hydrochloride (CPH) on this system, a “signal-off” type sensor was constructed. The sensor demonstrated excellent sensitivity and linear response to CPH concentrations ranging from 1 pmol L^−1^ to 100 μmol L^−1^, with a low detection limit of 0.4 pmol L^−1^ (S/N = 3).

## 1. Introduction

As a novel sensing platform, electrochemiluminescence (ECL) technology has gradually attracted tremendous attention due to its controllability, low background interference, high sensitivity, etc. [1,2]. Having an efficient ECL signal probe is critical to constructing a highly sensitive ECL sensor because it determines the output signal of the target [3,4]. It is well-known that graphitic carbon nitride (g-C_3_N_4_) has excellent film-forming ability and biocompatibility. Nevertheless, the ECL activity of the pristine g-C_3_N_4_ is much lower and cannot meet the requirements of the trace target [5,6]. In general, S_2_O_8_^2−^ is a co-reactant that can enhance the ECL intensity of g-C_3_N_4_ [7], while the use of high concentrations of co-reactants restricts further implementation in the ECL bioassay [8]. Fortunately, the co-reaction accelerator, which can accelerate the electroreduction process of S_2_O_8_^2−^ and implement the S_2_O_8_^2−^ in low concentrations was electroreduced to produce abundant SO_4_^•−^ and was introduced into the g-C_3_N_4_/S_2_O_8_^2−^ binary system to enhance the luminous efficiency [9,10]. Up to now, a series of nanomaterials with good catalytic activity for S_2_O_8_^2−^ have been reported, such as Ag NPs [11], CeO_2_ [12], CuS [13], and NH_2_-MIL(Fe) [14], which greatly improved the luminous efficiency of the luminophores in the ECL system. Among them, MOFs-based materials have become ideal candidate materials for co-reaction accelerators in ECL systems due to their excellent catalytic properties [15,16].

**Scheme 1:**
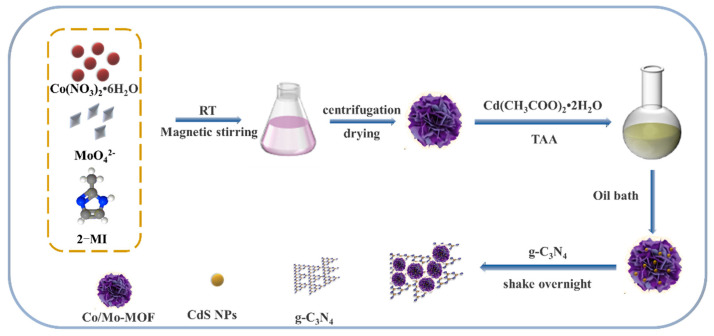
The schematic for the preparation of the material.

Notably, MOFs can be designed with two-dimensional (2D) structures, which exhibit excellent electronic properties, large specific surface area, and a high surface–volume atomic ratio [17]. More importantly, they possess many highly accessible active sites on the surface that are significant for applications in electrocatalysis and electrosensing [18,19]. Various kinds of ultrathin 2D MOFs have been explored, for example, MOF-74(Cu) [19], Zn-BTC MOFs [20], Ni/Fe-MOF [21], and Co/Ni-MOF [22]. Interestingly, Zeolitic imidazolate framework-67 (ZIF-67) is a self-assembled structure that was coordinated by Co^2+^ and 2-methylimidazole (2-MI), which offers exceptional flexibility in terms of morphological engineering and compositional control [14,23,24]. Furthermore, it possesses a 2D leaf-like structure synthesized via the aqueous solvent system [25,26]. Nevertheless, ZIF-67 still suffered from poor electrical conductivity and water stability, which greatly limited its applications as an electrocatalyst [27]. Specifically, it has been observed that the distinctive three-dimensional (3D) structures made of large-surface-area 2D nanosheets enhance electrochemical catalytic performance by reducing the electron diffusion distance and speeding up mass transfer [28]. Moreover, the design of efficient catalyst MOFs by molecular binders is a simple and novel approach [29]. Inspired by Ahn et al. [29], we synthesized a stereoscopic two-dimensional metal–organic framework with (Mo-O_4_)^2−^ as the molecular binder. Furthermore, CdS NPs, a widely used semiconductor material, have drawn a lot of attention [30]. The ECL strength and stability of CdS NPs@g-C_3_N_4_ were found to be better than that of pure g-C_3_N_4_ due to CdS NPs direct bandgap of 2.4 eV, which is near to that of g-C_3_N_4_ (2.7 eV) [31,32]. Intending to develop the co-reaction accelerator with high stability and efficiency and obtain the strong and stable ECL signal of g-C_3_N_4_, we have selected the 2D Co/Mo-MOF with mesoporous and fabricated CdS NPs-decorated Co/Mo-MOF composites, hereafter named CdS@Co/Mo-MOF (Scheme 1), for electrocatalytic S_2_O_8_^2−^ to generate SO_4_^•−^ and improve the ECL activity of g-C_3_N_4_. On the one hand, the presence of (Mo-O_4_)^2−^ in Co/Mo-MOF not only could induce the transformation of the oxidation state of Co ions but also participated in the coordination reaction of Co^2+^ along different directions, which promoted the formation of mesoporous structure [29,33], thus improving the electrocatalytic activity for S_2_O_8_^2−^ [34]. On the other hand, the loading of CdS NPs alleviated its aggregation and enhanced the performance of Co/Mo-MOF based on the synergistic role. Meanwhile, CdS@Co/Mo-MOF effectively alleviated the instability of the ECL signal of g-C_3_N_4_ [31], which effectively improved the accuracy of the detection of biological molecules.

Chlorpromazine hydrochloride (CPH) is a phenothiazine derivative containing the tertiary amine group, as well as a typical antipsychotic in clinical practice [35]. As an effective central dopamine receptor blocker, CPH has a significant effect on the treatment of acute and chronic mania, schizophrenia, bipolar disorder, tetanus, and vomiting [36]. However, excessive use of CPH can cause irreversible side effects on the human body, such as a decreased seizure threshold, decreased white blood cell count, tardive dyskinesias, and neuro malignant syndrome [37]. More importantly, when the content of CPH in human blood reaches 5–10 mg/mL, it can be life-threatening. Hence, it is crucial to develop a highly sensitive, stable, and reproducible method for the detection of CPH.

Inspired by the above, the depletion of core active substance sensing strategies for the detection of CPH was first developed based on CdS@Co/Mo-MOF as a co-reaction accelerator and g-C_3_N_4_ as a luminophore. Obviously, with the CdS@Co/Mo-MOF, the ECL emission of g-C_3_N_4_ was increased by 6.2-fold while stabilizing the ECL signal. As illustrated in Scheme 1, CdS@Co/Mo-MOF could accelerate the electroreduction of S_2_O_8_^2−^ to produce abundant SO_4_^•−^. Based on the reducibility of ^•^CPH [38], which induced the competitive reaction of CPH for SO_4_^•−^ during the ECL reaction, a solid-state ECL sensor was constructed for the sensitive determination of CPH.

## 2. Experimental Section

### 2.1. Chemicals and Materials

Ammonium molybdate tetrahydrate ((NH_4_)6Mo_7_O_24_·4H2O) was purchased from Shanghai Aladdin Reagent Co., Ltd. (Shanghai, China). Chlorpromazine hydrochloride (CPH), 2-methylimidazole (2-MI), cadmium acetate ((CH_3_COO)_2_Cd·2H_2_O), thioacetamide (TAA), and L-arginine (Arg) were ordered from Shanghai Macklin Biochemical Technology Co., Ltd. (Shanghai, China). Sodium carbonate (Na_2_CO_3_) was obtained from Chongqing Chuandong Chemical (Group) Co., Ltd. (Chongqing, China). All reagents were AR-grade and used without further purification. They were diluted with ultrapure water, obtained from a Millipore water purification system (18.2 MΩ·cm, Millipore, Burlington, MA, USA).

### 2.2. Preparation of g-C_3_N_4_

The synthesis of g-C_3_N_4_ was carried out using the thermo polymerization method reported previously [39,40]. Briefly, 5 g of the nitrogen-rich precursor melamine was added to a crucible with a lid. Then, it was placed in a muffle furnace and heated in air at an elevated rate of 5 °C/min until the temperature reached 550 °C and calcined for 4 h. The obtained bulk solid was ground into powder in a mortar. Finally, 1 mg/mL g-C_3_N_4_ suspension was prepared and then stripped by continuous ultrasonication for 24 h at a frequency of 40 kHz.

### 2.3. Preparation of Co/Mo-MOF

The preparation process of Co/Mo-MOF follows a previous report with some revisions [29,33]. First, 40 mL of Co(NO_3_)_2_⋅2H_2_O(40 mM) and 40 mL of 2-MI (120 mM) solutions were prepared and noted as solution A and solution B, respectively. Simultaneously, 20 wt% (NH_4_)_6_Mo_7_O_24_⋅4H_2_O (relative to 2-MI) was added to solution B and stirred for 3 h to fully bind the molybdate to the ligand. Subsequently, solution A was added to the above-mentioned solution under vigorous stirring and stirred for 2 h, and then maintained for 24 h. Finally, the Co/Mo-MOF was washed with ultrapure water, centrifugated three times (8000× *g* rpm, 4 °C, 5 min), and dried in an oven at 65 °C to obtain a light purple solid powder.

### 2.4. Preparation of CdS@Co/Mo-MOF

CdS@Co/Mo-MOF composites were synthesized according to a previous method [41,42] with some modifications. An amount of 48 mg of Cd(CH_3_COO)_2_⋅2H_2_O was uniformly dispersed in 100 mL of ethanol, and then 0.1 g of as-prepared Co/Mo-MOF was added and ultrasonicated for 30 min. Simultaneously, the suspension was stirred in an oil bath at 80 °C for 10 min. Subsequently, 13.8 mg of TAA was completely dissolved in 40 mL of deionized water and added drop by drop to the round bottom flask under vigorous stirring. The mixture was stirred at 80 °C for 30 min, and then the suspension was reacted at 80 °C for 30 min. The precipitate obtained by centrifugation was washed twice with ethanol and ultrapure water, respectively, and dried in an oven at 60 °C overnight. According to the weight ratio of CdS NPs in the composites, x%-CdS@Co/Mo-MOF (x = 10%, 30%, 40%, 60%) samples were prepared by adjusting the amount of Cd(CH_3_COO)_2_⋅2H_2_O and TAA.

### 2.5. Preparation of CdS NPs

CdS NPs were prepared by taking 0.24 g Cd(CH_3_COO)_2_⋅2H_2_O and 0.07 g TAA without the addition of Co/Mo-MOF, and the other conditions remained unchanged.

### 2.6. Preparation of CdS@Co/Mo-MOF@g-C_3_N_4_

Typically, 20 mg CdS@Co/Mo-MOF was dispersed in 10 mL ultrapure water, and then different volumes of as-prepared CdS@Co/Mo-MOF suspension were added to 2 mL 1 mg/mL g-C_3_N_4_ suspension. To prepare different ratios of CdS@Co/Mo-MOF@g-C_3_N_4_ ECL signal probe, the total amount of the prepared mixed liquid was controlled at 4 mL. After that, the resultant suspension was treated under ultrasonic conditions for 15 min. Subsequently, the mixture was placed in a shaker at 4 °C to shake overnight.

### 2.7. Preparation of M-ZIF-67, H-ZIF-67, and D-ZIF-67

The synthesis of ZIF-67 materials (M-ZIF-67, H-ZIF-67, and D-ZIF-67) was based on a previous report [25] with a slight improvement. Typically, M-ZIF-67 was synthesized with methanol as solvent, that is, 40 mL 40 mM Co(NO_3_)_2_⋅6H_2_O and 40 mL 120 mM 2-MI were prepared, respectively. Subsequently, these two solutions were mixed and stirred for 1 h and then aged for 24 h. Finally, the obtained purple precipitate was washed with methanol three times and dried in an oven at 65 °C overnight. The synthesis of H-ZIF-67 and D-ZIF-67 was similar to that of M-ZIF-67, except that the solvent system for H-ZIF-67 was ultrapure water and that for D-ZIF-67 was a mixture of DMF and ultrapure water (V_DMF_:$V_{H_{2}O}$ = 1:1).

### 2.8. Fabrication of the ECL Sensor

Firstly, the GCE was polished using a 0.05 µm Al_2_O_3_ slurry and then sonicated for 2 min in ultrapure aqueous nitric acid (*v*:*v* = 1:1), ethanol (*v*:*v* = 1:1), and water, respectively. Finally, 5 µL CdS@Co/Mo-MOF@g-C_3_N_4_ was directly immobilized onto the prepared GCE and dried at ambient temperature.

### 2.9. ECL Detection of CPH

ECL assays were performed in 0.1 M PBS containing 7 mM K_2_S_2_O_8_ and varying concentrations of CPH using an MPI-E type ECL workstation (Xi’an Remex Analytical Instrument Co., Ltd., Xi’an, China). All ECL tests were performed in a three-electrode system (Pt wire as the auxiliary electrode, saturated potassium chloride solutions electrode as the reference electrode, and GCE as the working electrode). The potential scanning range was −1.5–0 V, the scanning rate was 0.2 V/s, and the photomultiplier tube (PMT) was set to 500 V.

## 3. Results and Discussion

### 3.1. Characterization of the Different Nanomaterials

**Figure 1:**
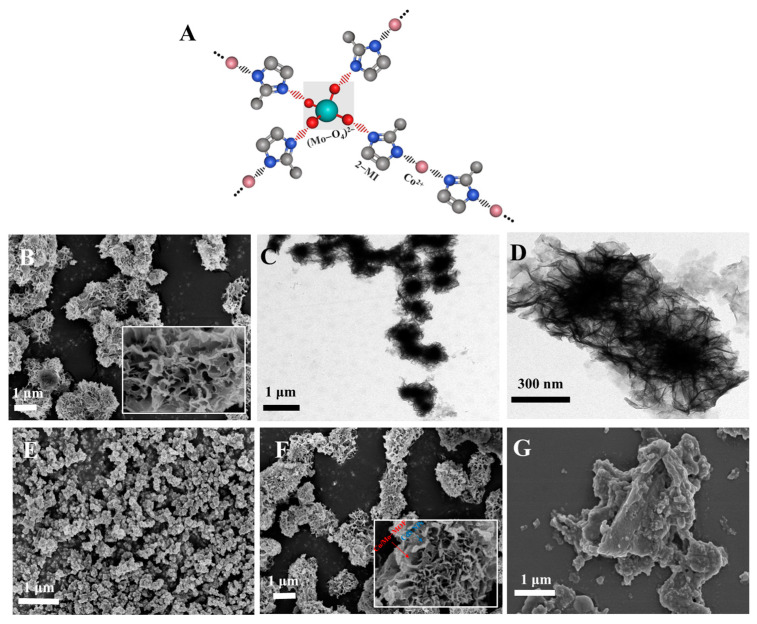
(**A**) Coordination process, (**B**) SEM image, and (**C**,**D**) TEM images of Co/Mo-MOF. And SEM images of (**E**) CdS NPs, (**F**) CdS@Co/Mo-MOF, and (**G**) g-C_3_N_4_.

Firstly, SEM and TEM were used to study the morphology of the prepared materials. Figure 1A shows the coordination process of Co/Mo-MOF, briefly, (Mo-O_4_)^2−^ as molecular binder bound to 2-methylimidazole ligand, and then the 2-MI-(Mo-O_4_)^2−^-2-MI assembly coordinated with Co^2+^ and participated in the coordination reactions of Co^2+^ along different directions, making the Co/Mo-MOF have a unique 3D structure assembled by nanosheets [33]. The SEM (Figure 1B) and TEM (Figure 1C,D) of Co/Mo-MOF further confirmed the coordination process. Specifically, Co/Mo-MOF was visible in the SEM image and TEM images, where the diameter was about 500 ± 50 nm with a flower-like structure assembled by nanosheets, which is consistent with the above speculation. As shown in Figure 1E, pure CdS NPs were easy to aggregate. It can be observed from Figure 1F that the granular CdS NPs were uniformly embedded on the surface of the Co/Mo-MOF. Importantly, the close contact between CdS NPs and Co/Mo-MOF facilitated the formation of heterojunction [41], which promoted the transfer and separation of charge. The SEM image of g-C_3_N_4_ showed an irregular lamellar structure (Figure 1G), demonstrating that the bulk g-C_3_N_4_ could be effectively stripped by long-term ultrasound.

**Figure 2:**
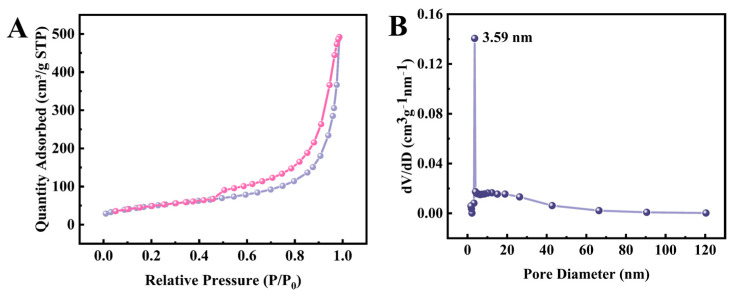
(**A**) N_2_ adsorption/desorption isotherms and (**B**) pore size distribution plot of Co/Mo-MOF.

**Figure 3:**
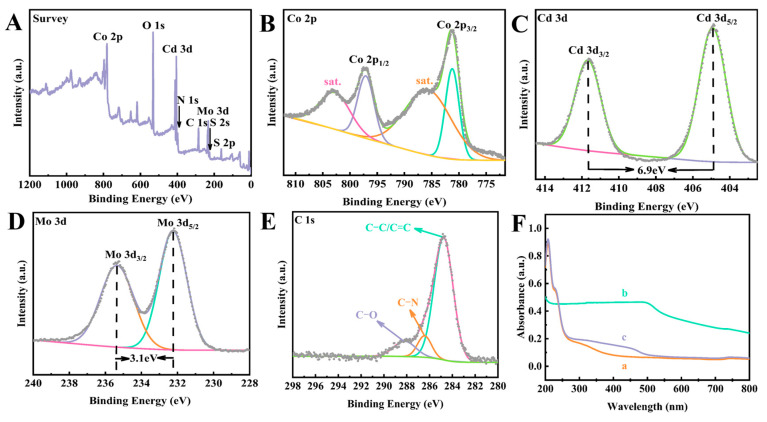
(**A**) Survey XPS spectrum of the CdS@Co/Mo-MOF, and high-resolution XPS spectra of (**B**) Co 2p, (**C**) Cd 3d, (**D**) Mo 3d, and (**E**) C 1s. And (**F**) UV–vis spectra of different materials (Co/Mo-MOF (a), CdS (b), and CdS@Co/Mo-MOF (c)).

Furthermore, the specific surface area and pore diameter of the Co/Mo-MOF were measured (Figure 2). The specific surface area and average pore size of Co/Mo-MOF are 176.364 m^2^/g and 13.097 nm, respectively, indicating that it has a mesoporous structure. Meanwhile, UV–vis spectrum and XPS further confirmed the successful synthesis of CdS NPs on Co/Mo-MOF nanosheets. As depicted in Figure 3F, for pure Co/Mo-MOF, the UV–vis absorbance peak appeared at about 210 nm and a shoulder absorption band appeared at 228 nm (curve a), while CdS NPs showed a wide absorption band at 200–500 nm (curve b). Compared with curve a, when Co/Mo-MOF was loaded with CdS NPs (curve c), the characteristic absorption of Co/Mo-MOF still existed, and the characteristic absorption band of CdS NPs became weaker, which confirmed that CdS NPs were successfully incorporated with Co/Mo-MOF nanosheets [41]. The XPS spectra of the CdS@Co/Mo-MOF further confirmed it. As shown in Figure 3A, the characteristic peaks of Co 2p (781.08 eV), O 1s (531.08 eV), Cd 3d (404.90 eV), N 1s (398.08 eV), C 1s (284.79 eV), Mo 3d (232.22 eV), and S 2p (162.08 eV) could be observed. Figure 3B shows the high-resolution spectra of Co 2p, the peaks located at 797.15 eV, and 781.04 eV were attributed to Co 2p_1/2_ and Co 2p_3/2_, respectively, and the peaks located at 803.38 eV and 785.88 eV corresponded to their satellite peaks, revealing that the valence state of Co in CdS@Co/Mo-MOF is +2 [43]. The high-resolution spectrum of Cd 3d (Figure 3C) displayed two sharp peaks located at 404.88 eV and 411.53 eV, confirming that Cd existed in the + 2 oxidation state [44,45]. In the Mo 3d region (Figure 3D), the peaks located at 232.23 eV and 235.38 eV corresponded to Mo 3d_5/2_ and Mo 3d_3/2_, respectively, and Mo was +6 valent [46,47]. Moreover, the high-resolution spectrum of C 1s (Figure 3E) exhibited the strongest peak at 284.73 eV, which was attributed to the α-carbon atom (C-C, C-H, or C=C) in the imidazole ligand. The peak at 286.42 eV corresponded to C-N, and the peak at 288.08 eV corresponded to C-O [48,49]. Among them, the peak at 288.08 eV might be attributed to the unwashed raw material during the in situ synthesis of CdS NPs.

### 3.2. Possible ECL Mechanism of the CdS@Co/Mo-MOF/g-C_3_N_4_/S_2_O_8_^2−^ Ternary System

**Figure 4:**
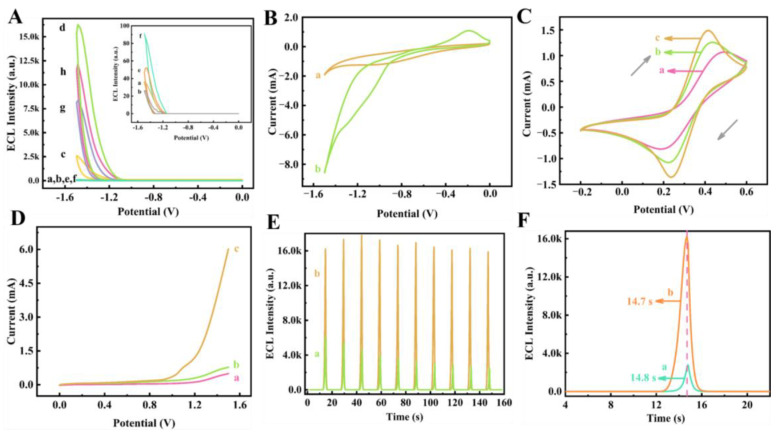
(**A**) The ECL responses of (a) GCE/S_2_O_8_^2−^, (b) g-C_3_N_4_, (c) g-C_3_N_4_/S_2_O_8_^2−^, (d) g-C_3_N_4_/CdS@Co/Mo-MOF/S_2_O_8_^2−^, (e) g-C_3_N_4_/CdS@Co/Mo-MOF, (f) CdS@Co/Mo-MOF/S_2_O_8_^2−^, (g) g-C_3_N_4_/H-ZIF-67/S_2_O_8_^2−^, and (h) g-C_3_N_4_/Co/Mo-MOF/S_2_O_8_^2−^. (**B**) The CV behaviors of various modified (bare GCE (a) and CdS@Co/Mo-MOF/GCE (b)) in 7 mM K_2_S_2_O_8_ solution. (**C**) The CV behaviors in 5 mM [Fe(CN)_6_]^3−/4−^ solution containing 0.1 M KCl and (**D**) LSV behaviors in PBS buffer of various modified (GCE (a), g-C_3_N_4_/GCE (b), and g-C_3_N_4_/CdS@Co/Mo-MOF/GCE (c)). (**E**) The ECL response curves of (a) g-C_3_N_4_/S_2_O_8_^2−^ and (b) g-C_3_N_4_/CdS@Co/Mo-MOF/S_2_O_8_^2−^ systems with consecutive 10 cycles CV scans. And (**F**) the peak position of the ECL signal of (a) g-C_3_N_4_ and (b) g-C_3_N_4_/CdS@Co/Mo-MOF.

To identify the luminophore and further reveal the ECL reaction mechanism of CdS@Co/Mo-MOF in the binary system g-C_3_N_4_/S_2_O_8_^2−^, ECL behaviors of different systems were tested and shown in Figure 4A. Specifically, the GCE/S_2_O_8_^2−^ system exhibited a weak signal response (curve a), corresponding to the ECL emission of singlet oxygen (^1^(O_2_)_2_^*^) [50]. The modified electrode (g-C_3_N_4_/GCE) in the PBS exhibited a weak cathode ECL emission (curve b), whereas the ECL signal was noticeably increased to 2613 a.u. in a solution containing 7 mM K_2_S_2_O_8_, which indicated that K_2_S_2_O_8_ was the co-reactant of g-C_3_N_4_ to improve the ECL response of the g-C_3_N_4_/S_2_O_8_^2−^ system (curve c). Interestingly, when CdS@Co/Mo-MOF was introduced into the g-C_3_N_4_/S_2_O_8_^2−^ binary system, the highest ECL emission of 16260 a.u. was obtained (curve d), which was about 6.2 times higher than that of the g-C_3_N_4_/S_2_O_8_^2−^ system. Importantly, the ECL response of CdS@Co/Mo-MOF@g-C_3_N_4_/GCE in PBS (curve e) was almost consistent with curve b, proving that CdS@Co/Mo-MOF could not enhance the ECL response by directly acting on g-C_3_N_4_. Curve f was the ECL response of the CdS@Co/Mo-MOF/S_2_O_8_^2−^ system, and a weak signal could be observed, indicating that the CdS@Co/Mo-MOF itself could not interfere with the ECL signal of g-C_3_N_4_. Meanwhile, the signal enhancement of the g-C_3_N_4_/K_2_S_2_O_8_ system by H-ZIF-67 and Co/Mo-MOF were investigated. The results showed that the ECL emissions of g-C_3_N_4_/K_2_S_2_O_8_/H-ZIF-67 and g-C_3_N_4_/K_2_S_2_O_8_/Co/Mo-MOF systems were 8350 a.u. (curve g) and 12,169 a.u. (curve h), respectively, which could be attributed to the unique structure of 2D Co/Mo-MOF and the synergistic effect between Co and Mo bimetals [33].

Similarly, the CV responses of GCE/S_2_O_8_^2−^ and CdS@Co/Mo-MOF/GCE/S_2_O_8_^2−^ were also recorded and are presented in Figure 4B. Specifically, a cathodic peak existed in curve a, which indicated the generation of SO_4_^•−^ by electroreduction of S_2_O_8_^2−^ under the cathodic potential scanning. In contrast, the current response of the cathodic peak corresponding to curve b was significantly enhanced and an oxidation peak appeared at −0.23 V, indicating that CdS@Co/Mo-MOF could accelerate the electroreduction process of S_2_O_8_^2−^. Therefore, the possible reaction mechanism of CdS@Co/Mo-MOF for signal amplification of the g-C_3_N_4_/S_2_O_8_^2−^ system could be described by Equations (1)–(8).

g-C_3_N_4_ + e^−^ → g-C_3_N_4_**^•−^** (1)

S_2_O_8_^2−^ + e^−^ → SO_4_^2−^ + SO_4_^•^^−^ (2)

Co^2+^ + S_2_O_8_^2−^ → Co^3+^ + 2SO_4_^2−^ + 2SO_4_^•^^−^ (more) (3)

(4) $${\mathrm{Co}}^{3+}+e^{-}\overset{{\left(\mathrm{Mo}-O_{4}\right)}^{2-}}{\to }{\mathrm{Co}}^{2+}\left(\mathrm{accelerate}\right)$$

g-C_3_N_4_**^•−^** + SO_4_^•^^−^ → g-C_3_N_4_* + SO_4_^2−^ (5)

or

g-C_3_N_4_ + SO_4_^•^^−^ → g-C_3_N_4_ + g-C_3_N_4_^+^ (6)

g-C_3_N_4_**^•−^** + g-C_3_N_4_^+^ → g-C_3_N_4_* + g-C_3_N_4_ (7)

finally,

g-C_3_N_4_* → g-C_3_N_4_ + hν (8)

In addition, to further investigate the electrochemical behavior of CdS@Co/Mo-MOF/g-C_3_N_4_/GCE, the CV and LSV measurements of bare and modified electrodes (g-C_3_N_4_/GCE, CdS@Co/Mo-MOF@g-C_3_N_4_/GCE) were carried out by the ECL workstation, respectively. In this case, the CV test was performed in 5 mM [Fe(CN)_6_]^3−/4−^ electrolyte containing 0.1 M KCl at a scan rate of 50 mV/s (Figure 4C). For bare GCE, a pair of symmetric redox peaks were observed (curve a) and ΔE_p_ (ΔE_p_ = E_pa_ − E_pc_) was 298 mV, indicating that the electron transfer kinetics on the surface of the electrode was slow. When g-C_3_N_4_ and CdS@Co/Mo-MOF/g-C_3_N_4_ were cast onto the bare GCE, the redox peak currents enhanced sequentially, and the ΔE_p_ decreased to 209 mV and 178 mV, respectively, indicating that the electron transfer kinetics on the surface of the electrode were improved. The LSV test was performed in 0.1 M PBS at a scan rate of 80 mV/s (Figure 4D) and its results showed the same electron transfer tendency as that of the CV tests. Specifically, compared with CdS@Co/Mo-MOF/g-C_3_N_4_/GCE, the current response of GCE and g-C_3_N_4_/GCE is lower, which could be attributed to the surface characteristics and high conductivity of CdS@Co/Mo-MOF@g-C_3_N_4_/GCE [51].

Interestingly, the ECL signal peak position of g-C_3_N_4_ modified with CdS@Co/Mo-MOF earlier 0.1 s than that of g-C_3_N_4_, showing better electron transfer performance (Figure 4F). Moreover, the ECL intensity–time curves that g-C_3_N_4_/S_2_O_8_^2−^ and g-C_3_N_4_/S_2_O_8_^2−^/CdS@Co/Mo-MOF systems were scanned for 10 cycles in the range of −1.5 V–0 V are shown in Figure 4E, indicating that CdS@Co/Mo-MOF had a certain signal stabilizing effect on the g-C_3_N_4_/S_2_O_8_^2−^ system. On the one hand, the direct band gap of CdS NPs (2.4 eV) is close to that of g-C_3_N_4_ (2.7 eV) [31]. On the other hand, the electron transfer mediated by the interface between CdS NPs and Co/Mo-MOF heterojunction alleviated the electrode passivation of g-C_3_N_4_ to some extent [41].

### 3.3. Investigation of the Electrochemical Differences

Due to the effect of polar solvents on nucleation and preferential crystal growth, we synthesized three types of ZIF-67 by varying the solvent. Among them, M-ZIF-67 was synthesized in methanol solvent; D-ZIF-67 was synthesized in a mixture of dimethylformamide (DMF) and ultrapure water (V_DMF_:$V_{H_{2}O}$ = 1:1); and H-ZIF-67 was synthesized in ultrapure water [25]. Cyclic voltammetry was used to investigate the electrocatalytic activity of ZIF-67/GCE. Compared with D-ZIF-67/GCE and M-ZIF-67/GCE, the redox peak current intensity of H-ZIF-67/GCE was the highest among the three types of ZIF-67 and showed a reversible reaction, displaying the best electrocatalytic property. Therefore, ultrapure water was used as the solvent of ZIF-67, thus synthesizing the 2D flower-like Co/Mo-MOF with a mesoporous in this work.

**Figure 5:**
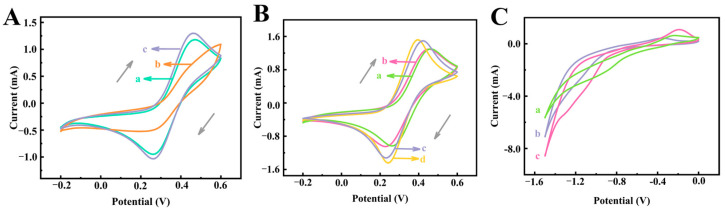
The CV behaviors of (**A**) ZIF-67 synthesized by the different solvent system (M-ZIF-67/GCE (a), D-ZIF-67/GCE (b), and H-ZIF-67/GCE (c)) and (**B**) various modified (H-ZIF-67/GCE (a), CdS NPs/GCE (b), Co/Mo-MOF/GCE (c), and CdS@Co/Mo-MOF/GCE (d)) in 0.1 M KCl solution containing 5 mM [Fe(CN)_6_]^3−/4−^. And (**C**) CV behaviors of different modified electrodes (H-ZIF-67/GCE (a), Co/Mo-MOF/GCE (b), and CdS@Co/Mo-MOF/GCE (c)) in 7 mM K_2_S_2_O_8_ solution.

To further explore the influence of molybdate ions doping and in situ composite CdS NPs on the electrochemical performance of H-ZIF-67, the CV behaviors of CdS@Co/Mo-MOF, Co/Mo-MOF, CdS NPs, and H-ZIF-67 were investigated, respectively, as well as the differences in the catalytic performance for K_2_S_2_O_8_ under applied voltage. Firstly, cyclic voltammetry (CV) is an approach for monitoring the changes in the surface features of electrodes. As shown in Figure 5B, the redox peak current intensity of CdS@Co/Mo-MOF (ΔE_p_ = 155 mV) was the highest compared to H-ZIF-67 (ΔE_p_ = 203 mV) and Co/Mo-MOF (ΔE_p_ = 189 mV), which might be the reason for molybdate ions doping as well as the synergistic effect of CdS NPs with Co/Mo-MOF. Next, the electrocatalytic activity of Co/Mo-MOF/GCE, H-ZIF-67/GCE, CdS NPs/GCE, and CdS@Co/Mo-MOF/GCE for K_2_S_2_O_8_ reduction were investigated by using the CV technique. The reduction peak current of the CdS@Co/Mo-MOF-immobilized GCE was more obvious than that of the H-ZIF-67- and Co/Mo-MOF-immobilized GCE in PBS solution containing 7 mM K_2_S_2_O_8_, indicating that CdS@Co/Mo-MOF has a stronger ability for the electroreduction S_2_O_8_^2−^. These results demonstrated that the properties of ZIF-67 could be effectively improved by synthesizing in the aqueous solvent system, doping with heterometallic elements, and in situ compounding CdS NPs.

### 3.4. Quenching Effect of CPH Towards the ECL Sensor

The quenching mechanism of CPH on the ECL sensor could be speculated as follows: the three six-membered rings in the phenothiazine nucleus in CPH were in the form of folding at certain angles. According to the molecular orbital theory, it had a higher orbital energy level and a stronger electron-donating ability. Moreover, the N atom of the phenothiazine ring was connected with a tertiary amine chain with strong electron-donating ability. Therefore, CPH was easier to lose electrons and formed a cationic radical intermediate ^•+^CPH [52]. The cationic radical intermediate ^•+^CPH lost its hydrogen ions to form the ^•^CPH active intermediate with a stronger reducing ability [53]. Due to the competitive reaction between the highly oxidizing SO_4_^•−^ and ^•^CPH, the g-C_3_N_4_^*^ obtained by an electron–hole recombination process might be hindered.

**Figure 6:**
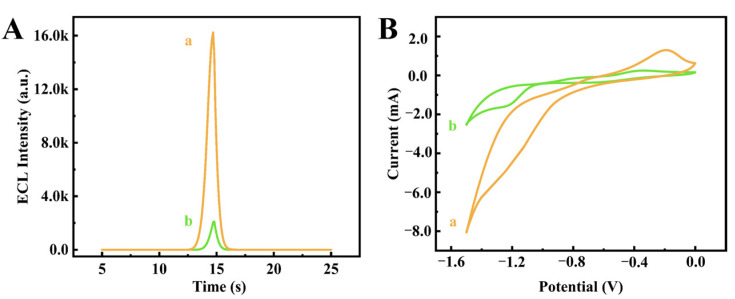
(**A**) ECL responses and (**B**) CV behaviors of different systems (without 1.0 mM CPH (a) and containing 1.0 mM CPH (b) in the detection solution).

In addition, ECL and CV analyses were employed to inspect the above speculation. Figure 6A demonstrates that CPH had a strong quenching effect on the ECL signal of this system. Figure 6B shows the CV behaviors with/without CPH in the detection solution. Compared with the CV curve without CPH (curve a), the reduction peak current of the CV curve containing CPH in the solution (curve b) was weakened, which further indicated that ^•^CPH consumes large amounts of SO_4_^•−^ [53]. In summary, SO_4_^•−^ does react with CPH, thus hindering the emission recombination of electron–hole trapped on the surface of g-C_3_N_4_, leading to the ECL quenching of the g-C_3_N_4_/S_2_O_8_^2−^/CdS@Co/Mo-MOF system. The process could be described by Equations (9)–(15).

CPH − e^−^ → ^•+^CPH (9)

CPH − H^+^ → ^•^CPH (10)

(11) CPH•→so4•−CPH+

g-C_3_N_4_^•^^−^ + SO_4_^•^^−^ (less) → g-C_3_N_4_* (less) + SO_4_^2−^ (12)

g-C_3_N_4_ + SO_4_^•^^−^ (less) → g-C_3_N_4_ + g-C_3_N_4_**^+^** (less) (13)

g-C_3_N_4_^•^^−^ + g-C_3_N_4_**^+^** (less) → g-C_3_N_4_* (less) + g-C_3_N_4_ (14)

finally,

g-C_3_N_4_* (less) → g-C_3_N_4_ + hν (15)

### 3.5. Optimization of Experimental Conditions

**Figure 7:**
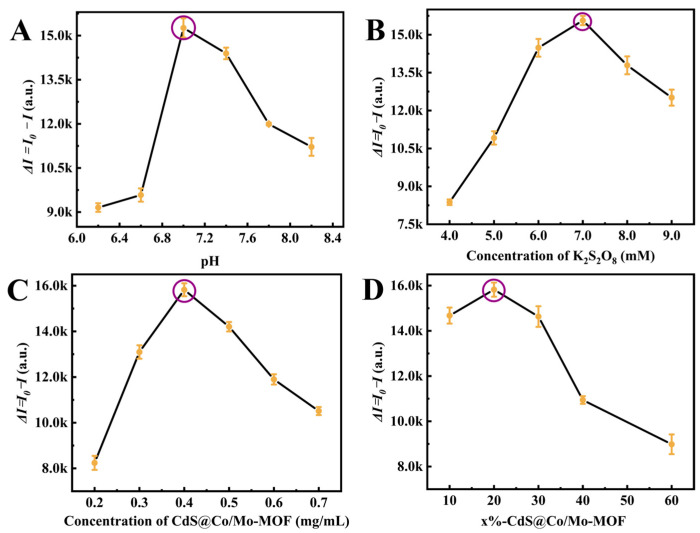
Effect of different experimental conditions on the response signals: (**A**) pH, (**B**) the concentration of K_2_S_2_O_8_, (**C**) the concentration of CdS@Co/Mo-MOF, and (**D**) x%-CdS@Co/Mo-MOF. Error bars: SD, *n* = 3.

To obtain a high-performance ECL sensor and accurately analyze the concentration of CPH, several important parameters affecting the optimal experimental results were investigated (Figure 7). Among them, *I* and *I*_0_ mean the ECL intensity of the presence and shortage of CPH, respectively, and Δ*I* = *I*_0_ − *I* was the difference. Firstly, the suitable environment of pH was crucial for the performance of the suggested sensor. As exhibited in Figure 7A, the ECL response of the biosensor was monitored in the pH range of 6.2–8.2, and the results showed that Δ*I* reached the maximum at pH 7.0. Subsequently, PBS with pH = 7.0 was used as the optimal experimental condition. Furthermore, K_2_S_2_O_8_ also had a great influence on the experimental results. As indicated in Figure 7B, the maximum Δ*I* appeared at 7 mM. Meanwhile, the concentration of CdS@Co/Mo-MOF nanosheets, which has a great impact on the luminous efficiency of the g-C_3_N_4_/S_2_O_8_^2−^ system, has been explored (Figure 7C). When the concentration of CdS@Co/Mo-MOF in the mixture exceeded 0.4 mg/mL or even higher, Δ*I* gradually decreased. In addition, to make the CdS@Co/Mo-MOF heterojunction composites have the best catalytic performance for S_2_O_8_^2−^, x%-CdS/Ni-MOF (x = 10%, 20%, 30%, 40%, 60%) with different CdS NPs loadings were prepared by adjusting the addition of Cd(CH_3_COO)_2_⋅2H_2_O and TAA and applied to the g-C_3_N_4_/S_2_O_8_^2−^ system (Figure 7D). The results showed that Δ*I* reached the maximum at x = 20%. As the loading of CdS NPs increased, the CdS NPs started to agglomerate, which blocked the electron transfer of Co/Mo-MOF [41], thus affecting the catalytic activity of the composites.

### 3.6. Analytical Performance of the Proposed ECL Biosensor

**Figure 8:**
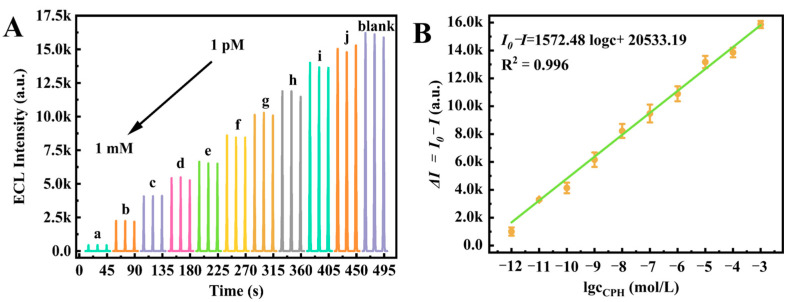
(**A**) The ECL response curves and (**B**) the linear calibration curve of the designed ECL sensor for a series of CPH concentrations.

To evaluate the property of the proposed sensor, the ECL intensity of different concentrations of CPH was investigated under optimal conditions. The behaviors of the g-C_3_N_4_-based ECL sensor at a series of CPH concentrations are exhibited in Figure 8A.

In Figure 8B, the ECL quenching intensity difference (*I*_0_ − *I*) shows a good linear relationship with the logarithm of CPH concentration (lgc) in the range from 1.0 × 10^−12^ M to 1.0 × 10^−5^ M. The linear equation can be expressed as *I*_0_ − *I* = 1572.48 lgc_CPH_ + 20,533.19 (R^2^ = 0.996). The limit of detection (LOD) was estimated to be 0.4 pmol/L based on the following Equation (16):

LOD = 3σ_b_/k (16)

where K represents the slope of the calibration curve, and σ_b_ denotes the standard deviation of three blank signals. Each experiment was conducted in triplicate [54]. Furthermore, the system demonstrated superior performance compared to many other CPH detection methods (Table S1 [55,56,57,58,59,60]), exhibiting significantly lower detection limits and an expanded detection range. Thus, this strategy had great potential in the quantitative detection of CPH.

### 3.7. Performance of the Proposed Biosensor

**Figure 9:**
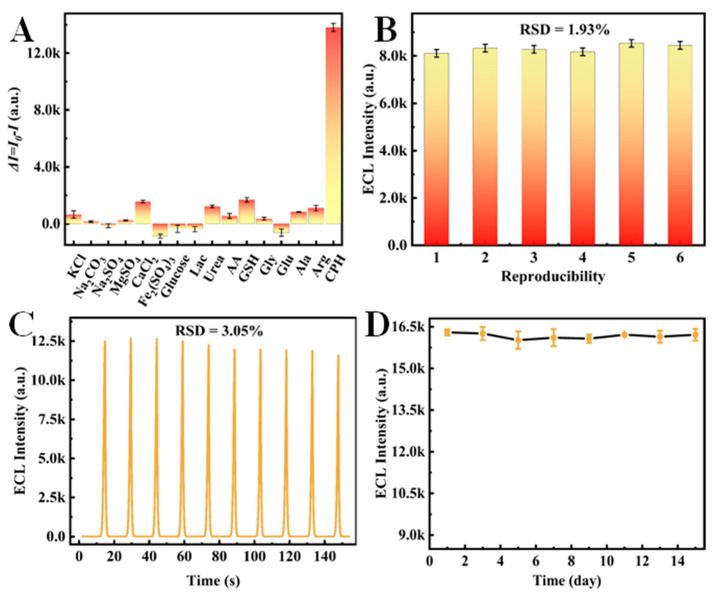
(**A**) Selectivity, (**B**) reproducibility, and (**C**) stability of the sensor. (**D**) The stability of the luminescent signal enhancement of CdS@Co/Mo-MOF composites in aqueous solution for 15 consecutive days.

To evaluate the selectivity of the ECL sensor, several interferences including metal ion compounds, amino acids, and biomolecules (i.e., KCl, Na_2_CO_3_, Na_2_SO_4_, MgSO_4_, CaCl_2_, Fe_2_(SO_4_)_3_, Glucose, Urea, Lactose, Arg, Glu, Gly, Ala, GSH and AA) were investigated under the same conditions (Figure 9A). Among them, the ECL responses in the presence and absence of interferences/CPH in the detection solution were expressed as *I* and *I*_0_, respectively, and the difference in quenching intensity was denoted by Δ*I* (Δ*I* = *I*_0_ *− I*). As presented in Figure 9A, compared with CPH (0.1 mM), the influence of these interferents (1.0 mM) on the ECL signal of the sensor was negligible, indicating that the sensor had good selectivity. Equally, the repeatability of the sensor was explored. Under the same conditions, six different modified electrodes were employed to determine 0.01 μM CPH (Figure 9B), and the relative standard deviation (RSD) was 1.93%, suggesting that the designed sensor has favorable reproducibility. Moreover, the stability of the biosensor was estimated under consecutive tests in the detection solution containing 0.1 nM CPH. As demonstrated in Figure 9C, the ECL signal was relatively stable, and the RSD was calculated to be 3.05%. Finally, the ECL signal probe (g-C_3_N_4_/CdS@Co/Mo-MOF) was dispersed in ultrapure water and recorded its ECL emission for 15 consecutive days to determine the water stability of CdS@Co/Mo-MOF (Figure 9D). Notably, the ECL intensity of this probe remained within a certain range, which suggested that the signal amplification of CdS@Co/Mo-MOF did not change significantly within 15 days in aqueous solution. To a certain extent, it showed that CdS@Co/Mo-MOF has good water stability.

### 3.8. Actual Sample Analysis

Further, the practical application of the sensor was evaluated by the standard addition recovery method. The ratio of the actual concentration (‘added’) to the detected concentration derived from the linear equation (‘found’) indicates the recovery. Firstly, the serum base fluid was prepared by centrifuging the healthy human serum and diluting 10-fold with 0.1 M PBS. Before the assay, the serum base solution was diluted 100-fold. Three parallel samples were obtained by adding a certain amount of CPH to the serum diluent. As shown in Table S2, when the CPH samples were added at concentrations of 0.1, 1, and 10 μmol/L, the recovery rates varied from 98% to 105%, and the relative standard deviation (RSD) ranged from 2.9% to 4.3%, indicating that the sensor could be applied to analyze the concentration of CPH in human serum.

## 4. Conclusions

In conclusion, CdS@Co/Mo-MOF was used as a novel and efficient co-reaction accelerator for amplifying the ECL signal of g-C_3_N_4_ and was applied for the first time in the fabrication of an ECL sensing platform for CPH determination. More importantly, the 2D flower-like CdS@Co/Mo-MOF with a mesoporous structure not only could enhance the cathodic ECL emission of g-C_3_N_4_ by speeding up the transformation of the S_2_O_8_^2−^ into SO_4_^•−^, but also alleviated the instability of the g-C_3_N_4_ signal. Notably, compared with the g-C_3_N_4_/S_2_O_8_^2−^ system alone, a 6.2-fold enhancement and stability ECL signal was obtained. Based on the competitive reaction between SO_4_^•−^ and ^•^CPH, the constructed sensor exhibited a low detection limit (0.4 pmol/L), and a wide linear range (1.0 × 10^−12^ mol/L–1.0 × 10^−3^ mol/L) in CPH analysis. It is worth pointing out that the successful use of 2D CdS@Co/Mo-MOF heterojunction composite in this ECL system also inspired other applications of 2D mesoporous MOF materials for research in ECL methodology.

## Data Availability

Data are contained within the article.

## Supplementary Materials

The following supporting information can be downloaded at: https://www.mdpi.com/article/10.3390/bios14120586/s1.
