# Supplementary material for: 2D Flower-like CdS@Co/Mo-MOF as Co-Reaction Accelerator of g-C3N4-Based Electrochemiluminescence Sensor for Chlorpromazine Hydrochloride
Source: Biosensors (Basel). 2024 Dec 2;14(12):586. doi: 10.3390/bios14120586 (PMC11674348; doi:10.3390/bios14120586)
Supplement: Supplementary file 1 [file biosensors-14-00586-s001.zip › biosensors-3283440-supplementary.pdf]

**Table S1.** Comparison with the reported analytical methods of CPH.

| Detection method | Linear range | Detection limit | Ref. |
|---|---|---|---|
| Fluorescence | 7.5–100.0 μM and 100.0–1000.0 μM | 0.043 μM | [1] |
| CL | 0.05–10 μg/mL | 6 ng/mL | [2] |
| DPV | 0.5–1472 μM | 0.001 μM | [3] |
| i-t | 0.0012–163.30 μM | 0.11 nM | [4] |
| SWV | 0.8–3.4 μM | 2.436 nM | [5] |
| SERS | $1 \times 10^{-1} – 1 \times 10^{2}$ μg/mL | 89.1 ng/mL | [6] |
| ECL | $1\times10^{-12}–1\times10^{-3}$ mol/L | 0.41 pmol/L | This work |

**Table S2**. Determination of spiked recovery of CPH in human serum (n = 3).

| Samples | Added(μmol/L) | Founded(μmol/L) | Recovery(%) | RSD(%) |
|---|---|---|---|---|
| 1 | 0.100 | 0.098 | 98.4 | 2.9 |
| 2 | 1.000 | 1.048 | 104.8 | 3.4 |
| 3 | 10.000 | 9.869 | 98.7 | 4.3 |

[1] Zhang L, Qin J, Yang Q, et al. Cost-effective and facile fluorescent probes for label-free recognition of chlorpromazine hydrochloride and logic gate operation [J]. Journal of Photochemistry and Photobiology A: Chemistry, 2019, 382: 111918.

[2] Shi W, Yang J, Huang Y, et al. Ion-pair complex-based solvent extraction combined with chemiluminescence determination of chlorpromazine hydrochloride with luminol in reverse micelles [J]. Journal of Pharmaceutical and Biomedical Analysis, 2004, 36(1): 197-203.

[3] Shanmugam R, Ganesamurthi J, Chen T W, et al. Preparation and fabrication of porous-$Fe_2O_3$/carbon black nanocomposite: a portable electrochemical sensor for psychotropic drug detection in environmental samples [J]. Materials Today Chemistry, 2022, 25: 100982.

[4] Jenisha Daisy Priscillal I, Wang S F. Highly sensitive amperometric determination of chlorpromazine hydrochloride in blood serum sample employing antimony vanadate nanospheres as electrode modifier [J]. Microchemical Journal, 2023, 187: 108396.

[5] Purushothama H T, Arthoba Nayaka Y, Manjunatha P, et al. Electrochemical determination of Chlorpromazine using l-Cysteine modified carbon paste electrode[J]. Chemical Data Collections, 2019, 23: 100268.

[6] Chen R, Chen Q, Wang Y, et al. Ultrasensitive SERS substrate for label-free therapeutic drug monitoring of chlorpromazine hydrochloride and aminophylline in human serum [J]. Analytical and Bioanalytical Chemistry, 2023, 415: 1803-1815.
